# Impact of reminiscence therapy on the well-being among elderly residents at an old age homes in Chennai, India

**DOI:** 10.6026/9732063002001994

**Published:** 2024-12-31

**Authors:** Schwartzlin Vinolia Paul Sathiyanathan, Theranirajan Ethiraj, Shankar Shanmugam Rajendran, Venkatesh Madhan Kumar, Anbalagan Marudan, Balasubramanian Sri Vidhya, Gayathri Ellappan Karunakaran

**Affiliations:** 1 Department of Psychiatric Nursing, College of Nursing, Madras Medical College, Chennai, The TN Dr MGR Medical University, Chennai, Tamil Nadu, India; 2 Department of Paediatrics, Madras Medical College, The TN Dr MGR Medical University, Chennai, Tamil Nadu, India; 3 Department of Paediatric Nursing, College of Nursing, Madras Medical College, The TN Dr MGR Medical University, Chennai, Tamil Nadu, India; 4 Department of Psychiatry, Institute of Mental Health, Madras Medical College, The TN Dr MGR Medical University, Chennai, Tamil Nadu, India; 5 Department of Child Health Nursing, Madras Medical College, The TN Dr MGR Medical University, Chennai, Tamil Nadu, India

**Keywords:** Reminiscence therapy, hedonic well-being, old age homes, quality of life, elderly care

## Abstract

The growing aging population presents challenges for healthcare systems, with many elderly residents in old age homes experiencing loneliness and reduced autonomy. Reminiscence therapy, which involves recalling past experiences, shows promise in improving well-being and quality of life. Therefore, it is of interest to evaluate the effectiveness of reminiscence therapy over 4 weeks at an old age home in Kodambakkam, Chennai, using a pre-test and post-test design with 50 elderly participants. Results showed significant improvements, with well-being scores increasing by 12.17% and quality of life scores rising by 15.69%. Qualitative data revealed that engagement and family dynamics contributed to positive outcomes, while feelings of loneliness remained complex. The findings suggest that reminiscence therapy can enhance psychological well-being and should be incorporated more widely in elderly care.

## Background:

Globally, the aging population is increasing at an unprecedented rate. By the year 2050, the global population of those aged 60 years or older is projected to double, reaching an estimated 2.1 billion individuals [1]. This demographic transition presents substantial obstacles to social frameworks and healthcare systems as they endeavour to address the distinct requirements of elderly individuals [2]. The high incidence of age-related problems, such as chronic illnesses, restricted mobility and impaired cognitive function, emphasizes the need for focused treatments to enhance the quality of life for older individuals [3]. Hedonic well-being, referring to the experience of good emotions and satisfaction with life, is paramount in determining the overall quality of life for Older Adults [4]. Existing research indicates that a mere 20% of older persons residing in old age homes express significant levels of life satisfaction, underscoring the need for targeted intervention in this crucial domain [5]. Furthermore, their hedonic well-being is greatly influenced by physical health, degree of independence and the quality of social relationships. These elements can enhance life contentment and general psychological well-being [6]. In recent years, reminiscence therapy has gained recognition as a beneficial psychological intervention for enhancing the quality of life in older adults by facilitating the recall and exchange of personal historical events [7]. This therapy modality facilitates stress management, augments quality of life and strengthens the perception of personal identity and continuity among persons [8]. A comprehensive examination of research on recollection therapy showed that individuals who regularly participated in sessions reported a notable 30% rise in their life satisfaction levels and a substantial decrease in their symptoms of depression in comparison to those who did not undergo the therapy [9]. The daily experiences of those residing in elderly care facilities substantially impact their general physical and mental health. Research has demonstrated that conductive settings that encourage social engagement, offer purposeful activities and uphold personal independence can significantly improve the well-being of inhabitants [10]. Moreover, tailoring care to align with residents' cultural and historical heritage can further augment their experience, fostering a sense of inclusion and comprehension.

## Materials and Methods:

An explanatory sequential design, to investigate the effects of reminiscence therapy on the hedonic well-being and quality of life of older people residing at a selected old age facility in Kodambakkam, Chennai was completed using a mixed-methods methodology. The study duration is four weeks.

## Qualitative phase:

After the quantitative analysis, a phenomenological design was used to carry out unstructured interviews with 5 older people. This facilitated a more comprehensive investigation of their encounters at the elderly care facility and the individual effectiveness of recollection therapy.

A quasi-experimental design, one-group pre-test and post-test design were initially used during the quantitative phase. Fifty older persons, aged 60-80 years, proficient in Tamil and willing to participate, were chosen using a non-probability convenience sampling method from the pool of eligible participants. The exclusion criteria encompassed critically ill senior individuals, those with sensory impairments and those afflicted with chronic diseases. Measurements of the Ryff Scale of Psychological Well-being and the WHO-QOL BREF were used to get the quantitative results. Participants' psychological well-being and quality of life were evaluated using the Ryff Scale, a 5-point scale consisting of 18 items and the WHO-QOL BREF, also a 5-point scale inclusive of 26 items.

## Ethical considerations:

The Institutional Ethics Committee of Madras Medical College, Chennai, approved the investigation. All participants gave written informed consent and strict confidentiality was maintained throughout.

## Results:

## Sociodemographic characteristics of the study participants:

The study predominantly involved elderly individuals aged 71-75, constituting 42% of the participants. The majority (36%) had completed middle school education and practiced Hinduism (82%). A significant proportion of the participants were widows (58%) and 70% reported having no source of income. Most participants (66%) had resided in the facility for 2-5 years. Almost all participants (96%) reported sleeping between 6 and 8 hours per night. Diabetes mellitus was identified as the most common health issue, affecting 40% of the study population.

## Initial levels of hedonic well-being and quality of life:

Initial hedonic well-being among the participants was significantly low, with 86% reporting low levels and only 14% experiencing moderate levels. None of the individuals evaluated their hedonic well-being as high. Accordingly, 78% of the individuals characterized their quality of life (QOL) as poor, 22% as intermediate and none initially rated it as good QOL.

## Effectiveness of reminiscence therapy:

The participants' hedonic well-being and quality of life were significantly enhanced after implementing reminiscence therapy. A 12.17% increase in well-being scores was seen, rising from 38.13% in the pre-test to 50.30% in the post-test. Likewise, the quality of life (QOL) scores increased by 15.69%, ascending from 40.81% in the pre-test to 56.50% in the post-test (Table 1 & Table 2).

## Comparative analysis of pre-test and post-test results:

A significant decrease occurred in the levels of hedonic well-being, as the percentage of low well-being declined from 86% to 36% after the intervention. The levels of moderate well-being rose from 14% to 64%. Significantly, the average hedonic well-being score increased from 48.04 to 63.38. Regarding quality of life (QOL), the percentage of individuals with low QOL declined from 78% to 24%, while the percentage of participants with moderate QOL rose from 22% to 76%. Furthermore, the average quality of life score rose dramatically from 40.81 to 56.50 (t=145.40, p<0.001).

## Association of demographic variables with post-test Well-Being and QOL:

Post-intervention results showed an association between selected demographic variables and improved QOL and well-being. Participants aged 61-65 years who had resided in the facility for 2-5 years exhibited higher post-test QOL scores. Furthermore, participants in the same age group who were free from health issues showed elevated post-test well-being scores.

## Qualitative findings on old age home experiences:

The qualitative analysis uncovered several themes concerning the participants' experiences in the old age home. The themes included "Comparison to Home", which encompassed feelings of ambivalence and changes in routine; "Engagement," which highlighted the importance of creativity and entertainment in fostering interaction; and "Family," where themes such as abandonment were prominent. The "Contented" theme revealed satisfaction and feelings of loneliness and isolation.

## Integration of qualitative and quantitative findings:

This study highlighted significant improvements quantitative indicators (well-being and quality of life) and qualitative experiences after reminiscence therapy. Initially characterized by poor well-being and quality of life (QOL), the outcomes after the intervention showed significant enhancements, with well-being scores rising to 50.30% and QOL to 56.50%. Complementing these results, qualitative data revealed alterations in daily routines and emotional reactions to family dynamics, demonstrating reminiscence therapy's intricate and multifaceted influence on participants' lives in the old age home environment.

## Discussion:

## Impact of reminiscence therapy on hedonic well-being and quality of life:

The outcomes of this study affirm the efficacy of reminiscence therapy in significantly enhancing the hedonic well-being and quality of life among the elderly, consistent with previous research indicating positive psychological impacts of reminiscence interventions [11, 12]. These findings are pivotal, as they highlight a practical approach to mitigating the generally low well-being and poor quality of life observed among elderly participants at the outset of this study, where 86% reported low hedonic well-being and 78% described their quality of life as poor. The substantial improvements from the pre-test to the post-test, where hedonic well-being increased by 12.17% and quality of life by 15.69%, demonstrate the potential of reminiscence therapy to foster not only a greater sense of past life reflection but also enhance present life satisfaction. These results are similar to those found by Shin *et al.* [13] and Patidar *et al.* [14] further validating the robust nature of reminiscence therapy as a beneficial geriatric intervention for hedonic well-being and quality of life. Comparison of pre-test and post-test results indicate a significant improvement in participant conditions, with low hedonic well-being rates dropping from 86% to 36% and moderate well-being rates rising from 14% to 64%. This substantial change in levels of well-being is supported by the findings of Allen *et al.* [15] and Basnet *et al.* [16], whose research similarly documented considerable improvements in the well-being and quality of life among older individuals after comparable treatments. The association between demographic factors and enhanced post-test scores indicated that younger, healthier individuals (aged 61-65 years) who had been in the facility for 2-5 years exhibited superior results. These findings indicate that specific demographic variables, such as age and health condition, substantially impact the efficacy of recollection therapy. The results above are consistent with the findings of Park *et al.* [17] and Ronkainen *et al.* [18], emphasizing the importance of considering certain demographic traits when customizing treatments to maximize therapeutic responses. The study's qualitative findings illuminate the complex emotional and social dynamics within old age homes. Themes such as Comparison to Home, Engagement, Family and Contentedness reveal a spectrum of experiences ranging from feelings of abandonment and loss to expressions of creativity and personal fulfillment. These nuanced insights, similar to those found by Whear *et al.* [19] and Fuller *et al.* [20]; highlight the importance of addressing the psychological and environmental factors contributing to elderly well-being. Integrating quantitative improvements in well-being and quality of life with qualitative experiences provides a comprehensive understanding of how reminiscence therapy impacts elderly individuals holistically. This dual approach not only supports the statistical evidence of the therapy's effectiveness but also enriches our understanding of its impact on the participants' day-to-day experiences and emotional states, confirming the findings of Laidlaw *et al.* [21] and Tominari *et al.* [22].

## Limitations of the study:

This study's limitations include the small sample size and the non-probability convenience sampling method, which may limit the generalizability of the findings. The study's short duration and lack of a control group also hinder the ability to definitively attribute improvements in well-being and quality of life to reminiscence therapy alone.

## Conclusion:

Nurses play an important role in successfully implementing reminiscence therapy, which this study has shown to significantly improve the hedonic well-being and quality of life among the elderly among old age homes. By facilitating sessions that encourage the sharing of memories, nurses help enhance the emotional and psychological health of elderly individuals. The findings suggest that targeted interventions like reminiscence therapy are effective and necessary to address the prevalent issues of low well-being and poor quality of life observed in this population. The study underscores the importance of considering demographic influences when applying such therapies and highlights the need for continued research to optimize care strategies within these communities.

## Data availability statement:

Data related to this study's findings are available upon reasonable request from the corresponding author.

## Figures and Tables

**Table 1:** Comparison of pre and post-test level of Well-being score

**Assessments**			**Well-being score**	
	**Maximum score**	**Mean well-being score**	**Percentage of well-being score**	**Percentage of well-being gain score**
Pre-test	126	48.04	38.13%	12.17%
Post-test	126	63.38	50.30%	

**Table 2:** Comparison of pre and post-test level of quality of life score

**Assessments**			**QOL score**	
	**Maximum score**	**Mean QOL score**	**Percentage of QOL score**	**Percentage of QOL gain score**
Pre-test	100	40.81	40.81%	15.69%
Post-test	100	56.5	56.50%	
